# Supplementary material for: Comprehensive Annotation and Functional Exploration of MicroRNAs in Lettuce
Source: Front Plant Sci. 2021 Dec 24;12:781836. doi: 10.3389/fpls.2021.781836 (PMC8739914; doi:10.3389/fpls.2021.781836)
Supplement: Supplementary file 2 [file Data_Sheet_8.ZIP › GSM2230750.plot/Lsa-miR171b_Lsat_1_v5_gn_7_9261.1_1167_TPlot.pdf]

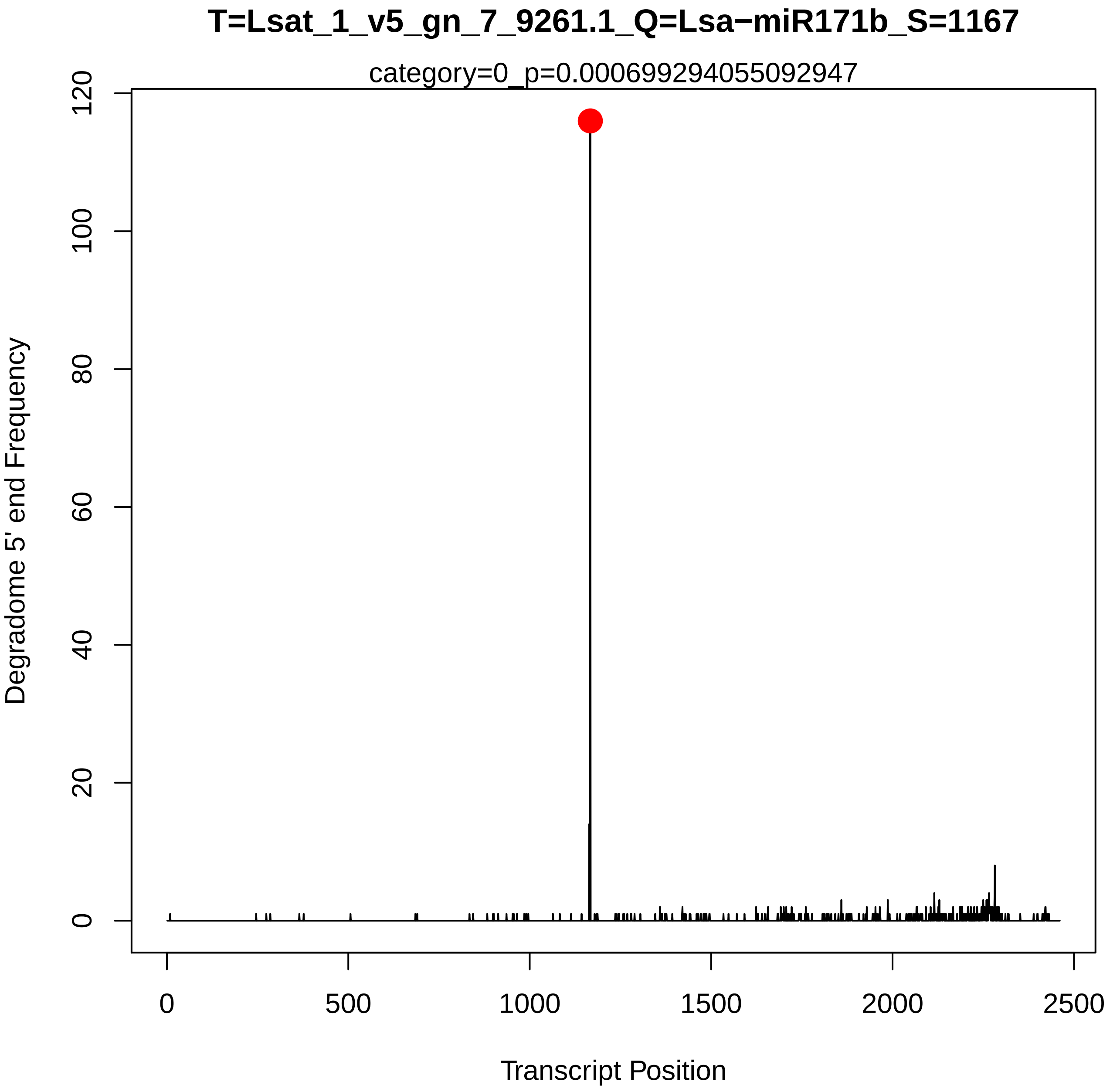
T=Lsat_1_v5_gn_7_9261.1_Q=Lsa−miR171b_S=1167
category=0_p=0.000699294055092947
Degradome 5' end Frequency
0
20
40
60
80
100
120
Transcript Position
0
500
1000
1500
2000
2500
